# 3D-FV-FE Aeroacoustic Larynx Model for Investigation of Functional Based Voice Disorders

**DOI:** 10.3389/fphys.2021.616985

**Published:** 2021-03-08

**Authors:** Sebastian Falk, Stefan Kniesburges, Stefan Schoder, Bernhard Jakubaß, Paul Maurerlehner, Matthias Echternach, Manfred Kaltenbacher, Michael Döllinger

**Affiliations:** ^1^Division of Phoniatrics and Pediatric Audiology, Department of Otorhinolaryngology, Head & Neck Surgery, Friedrich-Alexander-University Erlangen-Nürnberg, Erlangen, Germany; ^2^Institute of Fundamentals and Theory in Electrical Engineering, Division Vibro- and Aeroacoustics, Graz University of Technology, Graz, Austria; ^3^Division of Phoniatrics and Pediatric Audiology, Department of Otorhinolaryngology, Munich University Hospital (LMU), Munich, Germany

**Keywords:** computational fluid dynamics, computational aero acoustics, glottal insufficiency, left-right asymmetry, posterior gap, simVoice (numerical larynx model)

## Abstract

For the clinical analysis of underlying mechanisms of voice disorders, we developed a numerical aeroacoustic larynx model, called *simVoice*, that mimics commonly observed functional laryngeal disorders as glottal insufficiency and vibrational left-right asymmetries. The model is a combination of the Finite Volume (FV) CFD solver Star-CCM+ and the Finite Element (FE) aeroacoustic solver CFS++. *simVoice* models turbulence using Large Eddy Simulations (LES) and the acoustic wave propagation with the perturbed convective wave equation (PCWE). Its geometry corresponds to a simplified larynx and a vocal tract model representing the vowel /a/. The oscillations of the vocal folds are externally driven. In total, 10 configurations with different degrees of functional-based disorders were simulated and analyzed. The energy transfer between the glottal airflow and the vocal folds decreases with an increasing glottal insufficiency and potentially reflects the higher effort during speech for patients being concerned. This loss of energy transfer may also have an essential influence on the quality of the sound signal as expressed by decreasing sound pressure level (SPL), Cepstral Peak Prominence (CPP), and Vocal Efficiency (VE). Asymmetry in the vocal fold oscillations also reduces the quality of the sound signal. However, *simVoice* confirmed previous clinical and experimental observations that a high level of glottal insufficiency worsens the acoustic signal quality more than oscillatory left-right asymmetry. Both symptoms in combination will further reduce the quality of the sound signal. In summary, *simVoice* allows for detailed analysis of the origins of disordered voice production and hence fosters the further understanding of laryngeal physiology, including occurring dependencies. A current walltime of 10 h/cycle is, with a prospective increase in computing power, auspicious for a future clinical use of *simVoice*.

## 1. Introduction

The human voice as a prerequisite for speech production is our most important tool to communicate with other people. Moreover, people heavily rely on oral communication in their professional life. Disorders of the ordinary communication system have severe consequences on concerned persons' employments and even on the whole economic system (Ruben, 2000). The phonatory process, the prerequisite for human speech, describes the production of the human voice and depends on various factors as age, gender, training, and health status (Titze, 2000; Aronson and Bless, 2009).

**Figure 1:**
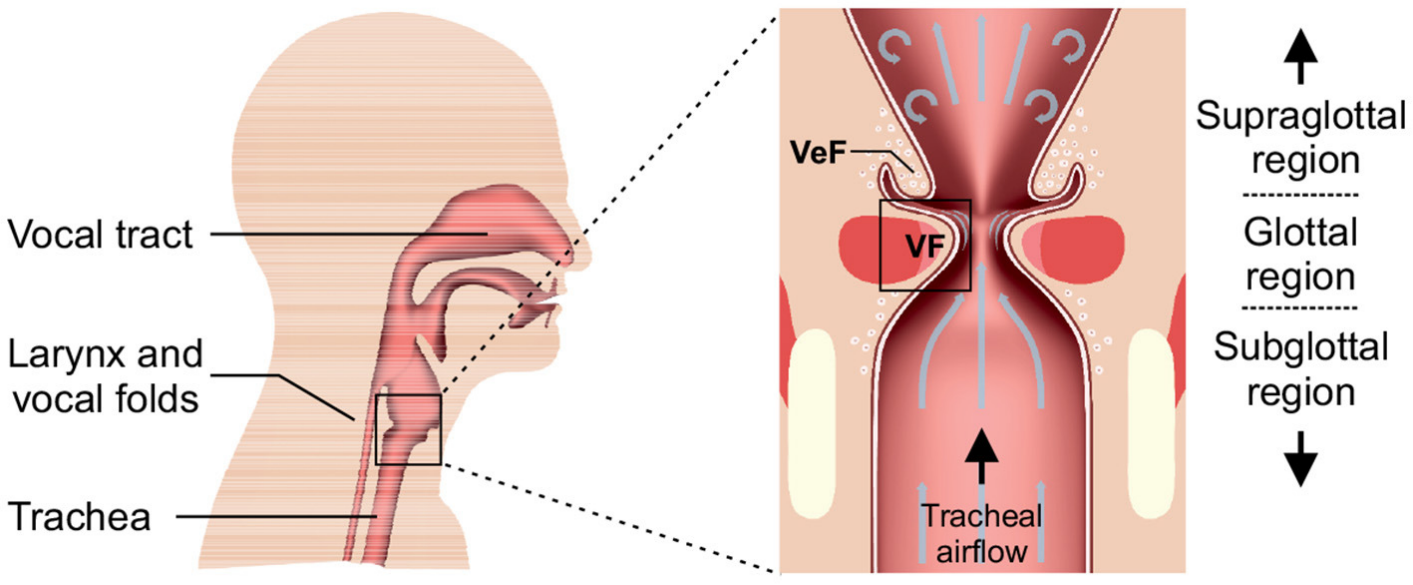
2D view of a human head **(left)** with an enlargement of the larynx **(right)** and its embedded structures that are important for the phonatory process. The vocal folds (VF) and the above arranged ventricular folds (VeF) are indicated.

The human voice results from a periodic oscillation of the vocal folds (VF) in the larynx, see Figure 1. The oscillations are caused by a complex fluid-structure interaction between the tracheal airflow and the elastic tissue of the vocal folds. Thereby, the airflow is the main sound generating source, that is subsequently modulated by the vocal tract consisting of the upper airway structures and is then emitted from the lips as an audible signal.

This process is supposed to be most efficient when (1) the vocal folds close the gap in between (called glottis) completely in each oscillation cycle and (2) when they oscillate symmetrically and periodic (Titze, 2000). An incomplete glottis closure or glottal insufficiency and asymmetric oscillations of the vocal folds cause a reduced voice quality with decreased tonal and increased broadband sound in the voice signal (Park and Mongeau, 2008; Hoffman et al., 2012; Yamauchi et al., 2016). The voice is then described as aspirated/breathy and hoarse. However, as shown by Inwald et al. (2010) and Schneider and Bigenzahn (2003), these underlying symptoms do not only occur in pathologic (e.g., scars, paresis, or paralysis) cases (Bhatt and Verma, 2014), but also in apparently organically healthy larynges (Rammage et al., 1992; Inwald et al., 2010; Patel et al., 2012) and with advancing age of the patients (Södersten et al., 1995; Vaca et al., 2017).

The scientific investigation and the clinical diagnostics suffer from the restricted location of the vocal folds inside the larynx, especially during phonation. To compensate this restriction, experimental (*ex/in vivo*), and numerical models have been developed. *In vivo* studies on glottal insufficiency were done by Södersten et al. (1995), Södersten and Lindestad (1990), and Yamauchi et al. (2014) and on the asymmetric vocal fold oscillations by Eysholdt et al. (2003). Whereas *in vivo* studies are difficult to perform and are mainly restricted to pure observation of the vocal fold oscillations (Inwald et al., 2010; Döllinger et al., 2012), *ex vivo* experiments with excised cadaver larynges (e.g., canine, porcine, human) provide better access to the laryngeal area and enable to manipulate the larynx (Hoffman et al., 2012; Birk et al., 2017b). *Ex vivo* studies about different levels of glottal insufficiency were reported by Döllinger et al. (2018) and Thornton et al. (2019) using rabbit larynges and Birk et al. (2017b) who used porcine larynges. Moreover, Oren et al. (2016) investigated asymmetric vocal fold oscillations in excised canine larynges.

Besides excised larynges, synthetic vocal fold models with silicone vocal folds were carried out with the focus on the glottal insufficiency (Park and Mongeau, 2008; Kirmse et al., 2010; Kniesburges et al., 2013, 2016). Pickup and Thomson (2009) and Zhang et al. (2012) investigated asymmetric vocal fold oscillations with a silicone model. Such models can mimic specific physiological and disordered motion patterns of the vocal folds for which they have been developed for and are therefore well-established in voice science (Zhang et al., 2004; Thomson et al., 2005; Park and Mongeau, 2008; Kirmse et al., 2010; Murray and Thomson, 2012; Kniesburges et al., 2013, 2016; Van Hirtum and Pelorson, 2017; Motie-Shirazi et al., 2019; Taylor et al., 2019; Romero et al., 2020). However, both *ex vivo* and synthetic larynx models are restricted regarding the spatial resolution of the measuring data of fluid flow, the vocal fold dynamics, and their interaction.

Thus, numerical models based on Finite-Elements and/or Finite-Volumes have great potential to be applied in the clinical routine, e.g., diagnostics and treatment control. Numeric simulations, regarding the effect of the glottal insufficiency on the human voice, were done by Zörner et al. (2016) and on the asymmetric vocal fold oscillations by Xue et al. (2010) and Samlan et al. (2014). In contrast to experimental models, computer models provide the complete 3D data of the flow field (Sciamarella and Le Quéré, 2008; Zörner et al., 2013; Sadeghi et al., 2018) and in case of coupled models the fluid-structure interaction (FSI) between flow, tissue and the aeroacoustic sound generation and propagation during phonation (de Oliveira Rosa et al., 2003; Luo et al., 2008, 2009; Tao and Jiang, 2008; Link et al., 2009; Kaltenbacher et al., 2014; Xue et al., 2014; Jo et al., 2016).

The large drawback of these numerical models are the large computational costs to perform the simulations (Sadeghi et al., 2018). Thus, they are not applicable in the clinical environment yet, where a short wall time with sufficient accuracy is needed. However, computational fluid dynamic (CFD) models with prescribed vocal fold movements and a prospective increasing computational power already keeps the simulation time adequately small (Sadeghi et al., 2019b).

For the development of our hybrid (sound propagation is calculated based on aeroacoustic source terms from the flow simulation) 3D aeroacoustic numeric larynx model *simVoice* (Sadeghi et al., 2018, 2019a,b; Schoder et al., 2020) for future clinic usefulness, it is essential to replicate normal and disordered glottal closures and dynamical asymmetries. A method to set up a workflow containing the import of various physiological and disordered glottal geometries into *simVoice* is shown in this study. We concentrate on modeling four disordered cases of glottal insufficiency based on high-speed video data of porcine *ex vivo* experiments performed by Birk et al. (2017b). Moreover, symmetric and asymmetric vocal fold motions are modeled. Our hypotheses for this study are:

- Hypothesis 1: Our existing and validated 3D-FV-FE numerical larynx model *simVoice* can accurately mimic and simulate realistic glottis geometries and vocal fold motions based on experimental high-speed video data.
- Hypothesis 2: *simVoice* can qualitatively and quantitatively mimic typical glottal parameters quantifying the different levels of glottal insufficiency that are reported in the literature.
- Hypothesis 3: Typical parameters of the acoustic voice signal computed from the simulated sound signal show typical characteristics for glottal insufficiency and asymmetric vocal fold oscillations.

## 2. Methods: Hybrid Aeroacoustic Numerical Larynx Model—*simVoice*

The 3D aeroacoustic numeric larynx model *simVoice* is a combination of the Finite Volume (FV) CFD solver Star-CCM+ and the Finite Element (FE) solver CFS++ (Kaltenbacher, 2015). The basic *simVoice* model was validated against a silicone model that provided an extensively large set of experimental data, including the vocal fold motion, the flow field, and produced sound field (Kniesburges et al., 2013, 2016, 2020; Lodermeyer et al., 2015, 2018). Characteristic parameters of the silicone model performance and corresponding physiological male values are shown in Table 1. Validation parameters in detail were: (1) Flow dynamic properties as pressure measurements and the velocity field with the glottal jet in the supraglottal region using particle image velocimetry (PIV) by Sadeghi et al. (2018, 2019a), and (2) the acoustic signal by Schoder et al. (2020). In this study, the investigated configurations of glottal insufficiency and asymmetric vocal fold oscillations are synthetic cases that were derived as combination from *ex vivo* (Birk et al., 2016, 2017a) and silicone model experiments (Kniesburges et al., 2013). Thus, there are no experimental data for validation purposes.

**Table 1:** Parameter reported for normal male phonation in *in vivo* and *ex vivo* studies compared with the experimental silicone model *synthVOICE* (Kniesburges et al., 2013, 2016, 2020; Kniesburges, 2014) (validation cases) and the performed numerical validation simulations by *simVoice* (Sadeghi et al., 2018, 2019a,b; Sadeghi, 2019; Schoder et al., 2020).

**Parameter**	***In vivo* (male)**	***Ex vivo* (male)**	**Silicone model (*synthVOICE*)**	**Numerical simulation (*simVoice*)**
Fundamental Frequency (*F*_0_) [Hz]	103–220 (Larsson and Hertegård, 2004; Sundberg et al., 2005)	97–200 (Döllinger et al., 2005, 2016; Döllinger and Berry, 2006b)	148	148
Vocal fold length (anterior–posterior) [mm]	14–17 (Schuberth et al., 2002; Hoppe et al., 2003; Larsson and Hertegård, 2004; Rogers et al., 2014)	13–18 (Lagier et al., 2017)	15	15
Glottal gap diameter (*d*_*G*_) [mm]	1.49–2.8 (Hoppe et al., 2003; George et al., 2008; Semmler et al., 2018)	2.3–5.6 (Döllinger et al., 2005, 2016; Döllinger and Berry, 2006a,b; Boessenecker et al., 2007)	4.66	4.66
Speed Quotient (*SQ*) [a.u.]	0.59–1.978 (Holmberg et al., 1988; Baken and Orlikoff, 2000)	0.8–1.6 (Döllinger et al., 2014)	0.67	0.67
Open Quotient (*OQ*) [a.u.]	0.37–1.00 (Holmberg et al., 1988; Baken and Orlikoff, 2000)	0.42–1.00 (Mendelsohn et al., 2015)	0.93	0.93
Mean flow rate ($\bar{Q}$) [$\frac{l}{min}$]	4.5–18 (Holmberg et al., 1988; Baken and Orlikoff, 2000)	6–108 (Döllinger et al., 2005, 2014, 2016; Döllinger and Berry, 2006a,b; Boessenecker et al., 2007)	65–115	37.8–132
Mean subglottal pressure (*P*_*sub*_) [Pa]	157–3510 (Holmberg et al., 1988; Sundberg et al., 1993, 2005; Alku et al., 2006)	600–4300 (Döllinger et al., 2005, 2014, 2016; Döllinger and Berry, 2006b)	2449–3251	2450–3251

### 2.1. *simVoice*—CFD

#### 2.1.1. Geometric Dimensions

**Figure 2:**
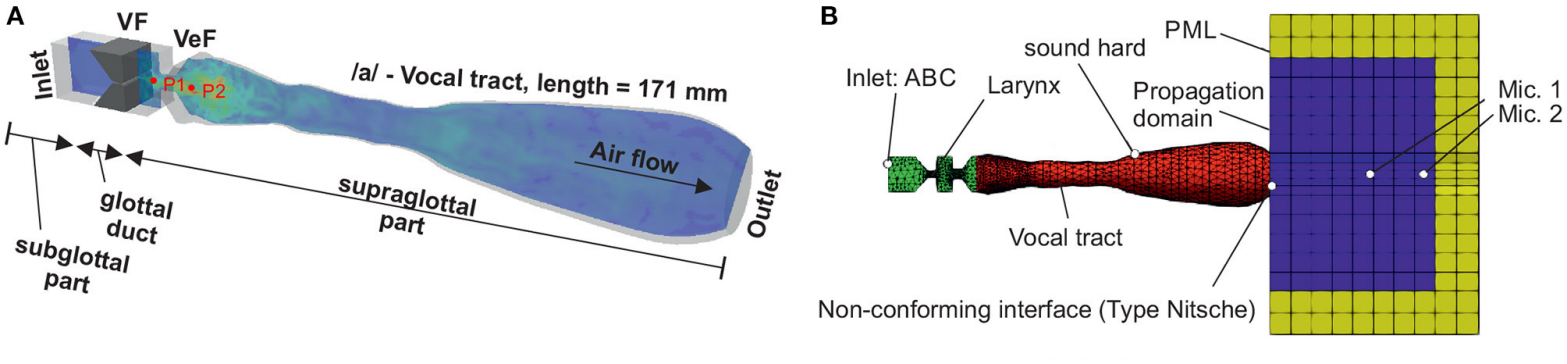
**(A)** 3D representation of *simVoice*, including a velocity field in the mid-coronal plane, the vocal folds (VF), the ventricular folds (VeF), and the vowel /a/-vocal tract. Points P1, and P2 are located 6 mm, and 20 mm in distance to the vocal folds. **(B)** Geometry and domain of the CAA model of *simVoice* as introduced by Schoder et al. (2020). Mic.1 and Mic.2 are located 5 and 8 cm in distance of the vocal tract exit (mouth).

The CFD model *simVoice* represents three main parts: the subglottal section upstream of the vocal folds, the glottal duct with the two vocal folds (VF) and the supraglottal part with the ventricular folds (VeF) and an MRI-based vocal tract (VT), see Figure 2A. The vocal folds are based on the well-known M5 model (Scherer et al., 2001; Thomson et al., 2005) and the numerical domain dimension is obtained from the experimental setup of a synthetic vocal fold model (Becker et al., 2009; Kniesburges et al., 2013, 2016; Lodermeyer et al., 2015). All dimensions of the larynx structures are in the human length scale (Titze, 2000). The basic development of *simVoice* is described in (Sadeghi et al., 2018, 2019a,b). The gap between the VeF is 5 mm as in (Sadeghi et al., 2019a). The vocal tract represents the vowel /a/ and was developed by Probst et al. (2019) based on MRI data of 6 professional tenors (Echternach et al., 2011). Probst et al. (2019) simplified the single tenors' VTs with the method introduced by Story et al. (1996) and generated a mean vocal tract model by averaging the six single vocal tracts. The resulting staged vocal tract model was subsequently smoothed with linear interpolation. Arnela et al. (2016) showed, that using a simplified vocal tract instead of a realistic vocal tract is an appropriate approach. The distance between the vocal folds and the outlet of the vocal tract is 171 mm.

#### 2.1.2. Modeling the Glottis Geometry

**Figure 3:**
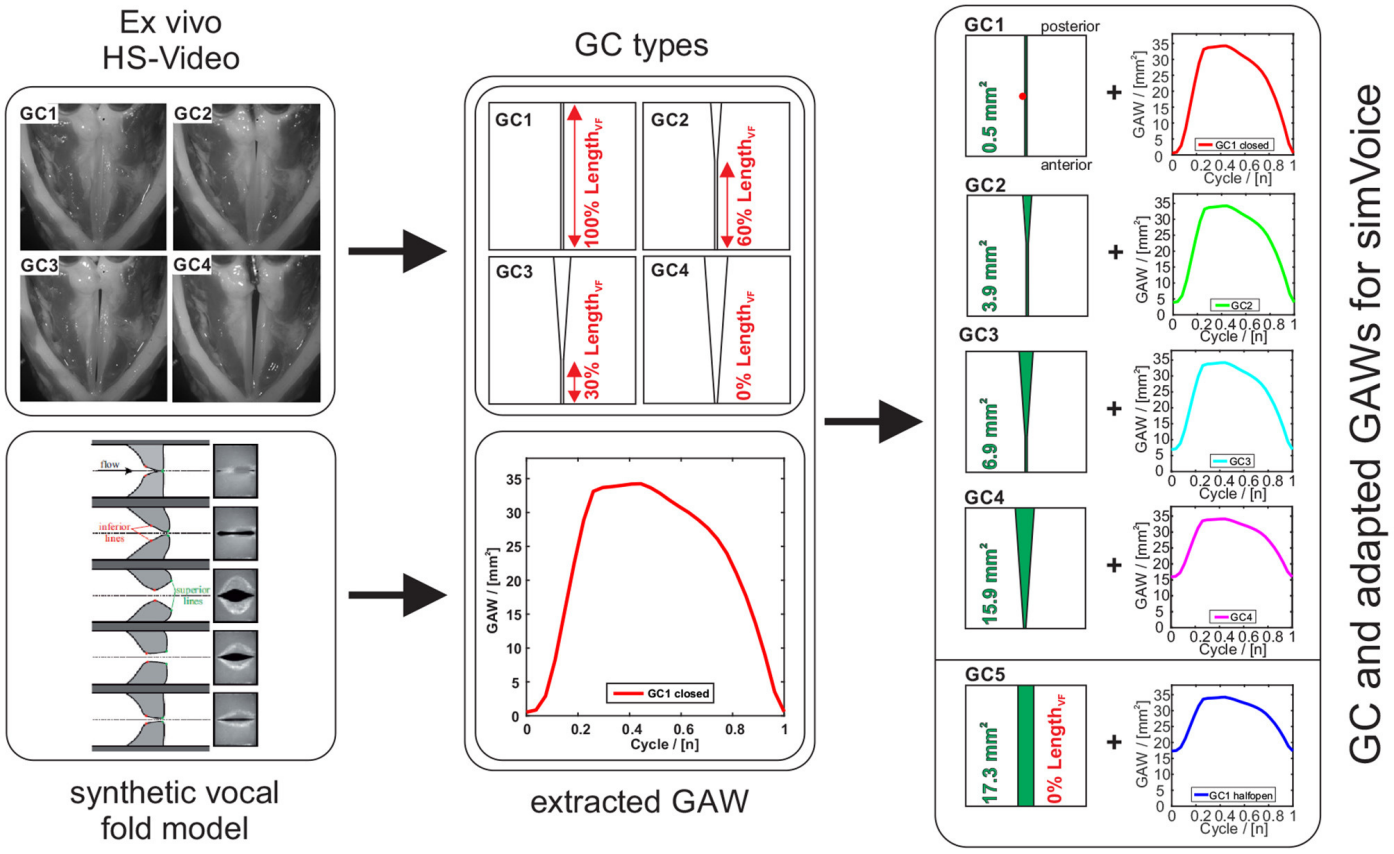
Workflow of vocal folds modeling. Upper left part: four GC types of *ex vivo* experiments based on high-speed videos (Birk et al., 2017b) and the corresponding schematic numeric GC geometries (superior view). Lower left part: phases of the vocal fold motion of the synthetic vocal fold model (view on the coronal plane) during one oscillation cycle (Lodermeyer et al., 2015) and the GAW was taken from high-speed videos (Sadeghi et al., 2018). Right part: Four plus one additional GC types with the adapted GAWs.

In this study, four types of clinically seen glottis closures (GC1 to GC4) were designed that are based on high-speed recordings obtained from experiments with *ex vivo* porcine larynges by Birk et al. (2016, 2017a), see Figure 3. Furthermore, an additional type GC5 with a rectangular glottis shape, similar to a midmembranous gap (Södersten et al., 1995), was modeled. GC1 to GC4 represent posterior gaps with an increasing glottal insufficiency, whereas GC5 represents a complete glottal insufficiency. A glottal insufficiency can not only occur in pathological phonation cases as a result of scars, paresis, paralysis, or age-related atrophy (Bhatt and Verma, 2014; Vaca et al., 2017), but also in physiological phonation of women or children with a triangular-shaped gap located at the posterior part of the glottis (Södersten and Lindestad, 1990; Rammage et al., 1992; Södersten et al., 1995; Inwald et al., 2010; Döllinger et al., 2012; Patel et al., 2012). All GC types are modeled by two parameters: (1) the initial glottal gap area and (2) the length of the closed part of the glottis divided by the entire glottis length. As shown in Figure 3, the modeled glottis is either fully closed (GC1: 100% *Length*_*VF*_), partly closed (GC2: 60% and GC3: 30% *Length*_*VF*_), or completely open (GC4 and GC5: 0% *Length*_*VF*_) at the initial glottal gap. The initial glottal gaps for GC2 to GC4 are based on the glottal gap index of Birk et al. (2016, 2017a) and the initial glottal gap of GC5 is half the maximum GAW of the synthetic model (Kniesburges et al., 2016). As described by Sadeghi et al. (2018), there must be a small area between both vocal folds of 0.5*mm*^2^ at GC1 to reach a numerically stable simulation. Nevertheless, this small gap still interrupts the flow through the glottis during the closed phase, as shown by Sadeghi et al. (2019b). For GC2, GC3, and GC4, the initial glottal gaps possess a triangular and for GC5 a rectangular shape, see Figure 3.

#### 2.1.3. Vocal Fold Motion

The lower part of Figure 3 shows the phases of the synthetic vocal folds during one oscillation cycle (Lodermeyer et al., 2015; Kniesburges et al., 2020) and the corresponding glottal area waveform (GAW). The GAW is computed as the change of glottal area over time and is a common measure for the description of laryngeal dynamics. Based on the GAW of the synthetic model (Kniesburges et al., 2016), the oscillation of the vocal folds is modeled in *simVoice* as proposed by Sadeghi et al. (2018). In the right part of Figure 3, the five GC types combined with the respective modified GAWs are shown. The GAW for GC1 is equal to that used by (Sadeghi et al., 2018). The GAWs for GC2 to GC5 were computed as follows:

(1) $$\begin{array}{r} A_{i}\left(t\right)=A_{i}^{0}+\frac{A_{0}^{max}-A_{i}^{0}}{A_{0}^{max}}\cdot A_{0}\left(t\right) \end{array}$$

where *A*_*i*_(*t*) is the modified GAW (of the individual GC type), subscript *i* = 0 indicates the GAW of the synthetic model of Kniesburges et al. (2016), and subscript *i* = 1 to 5 indicates GC1 to GC5. $A_{i}^{max}$ is the maximum value of the GAW and $A_{i}^{0}$ represents the initial glottal gap area, see Figure 3.

We explicitly selected one motion pattern in combination with the five increasing levels of glottal insufficiency (GC1-GC5). With this strategy, we avoided to include individual effects of the patient-specific motion that may overlap the effects of the glottal insufficiency in the acoustic results.

To reduce the computational costs of the CFD simulations, the vocal fold dynamics are externally forced with characteristic dynamic patterns according to the modified GAWs. The computation of the elliptic shaped vocal fold motion is generated by a sinusoidal function along the two vocal folds (Sadeghi et al., 2018). Additionally, Sadeghi et al. (2018) added a simple convergent-divergent standard mucosal wave-like motion model based on experiments (time periods of convergent and divergent glottal duct shapes) and the literature for typical angles of the glottal duct during oscillation (Titze, 2000). It contains a convergent shaped glottal duct during the opening (0.1 T to 0.32 T) with an angle range of 0° to 5° and a divergent duct (0.32 T to 0.9 T) with angles of −10° to 0°. The glottis is closed between 0.9 T and 0.1 T of the next cycle. The 3D vocal fold motion is realized by moving wall boundaries of the vocal folds that form the glottal duct, see Supplementary Video 1. For all GC types, the vocal folds oscillate with a fundamental frequency of *f*_0_ = 148*Hz*. The maximum glottis width of 4.66 mm is in the range as reported for *ex vivo* male larynx studies (up to 5.6 mm)(Döllinger et al., 2005; Döllinger and Berry, 2006a,b; Boessenecker et al., 2007) but higher than reported for *in vivo* measurements (up to 2.8 mm) (George et al., 2008; Semmler et al., 2018).

**Figure 4:**
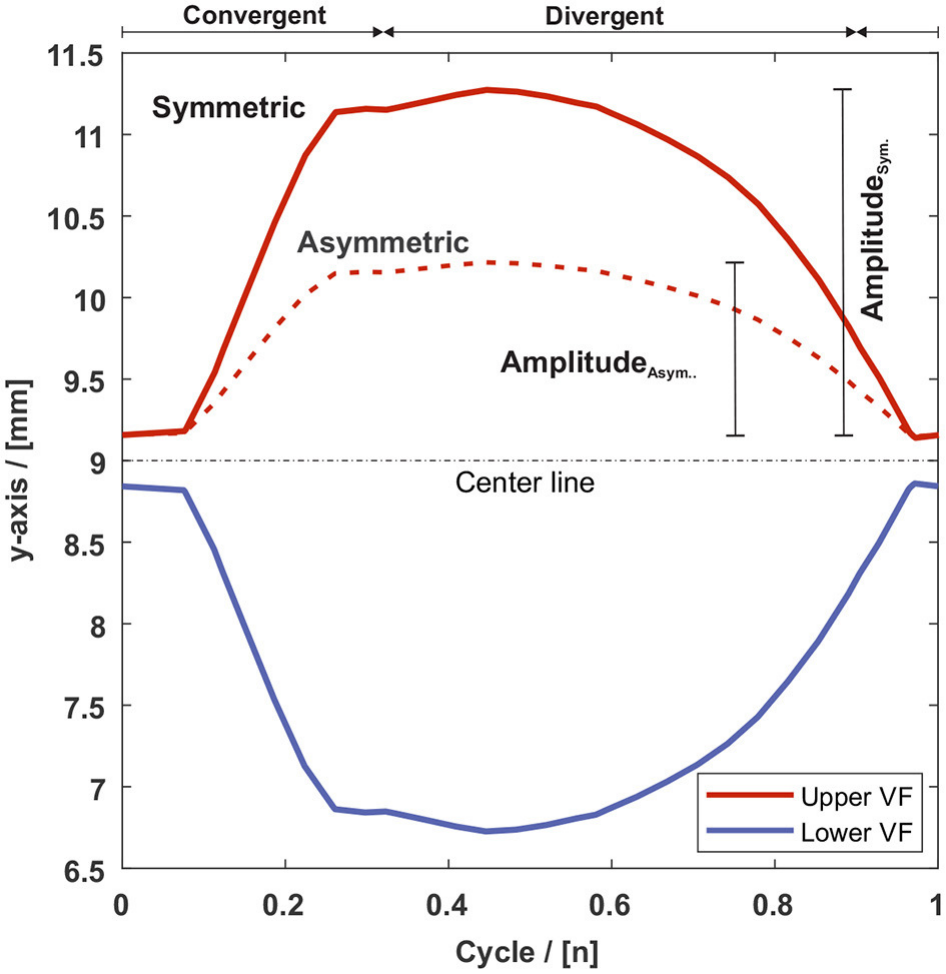
Exemplary vocal fold motion of GC1 for the symmetric and asymmetric case along the y-axis (medial-lateral direction) for a point on the medial plane of the VF surface, see the red mark at GC1 in Figure 3. The solid red line represents the motion on the y-axis of the upper vocal fold for the symmetric and the dashed line for the asymmetric motion. The blue line represents the motion on the y-axis of the lower vocal fold.

For the symmetric motion, both vocal folds move equally but in opposite directions. The left-right asymmetric vocal fold motion is realized by reducing the amplitude of one vocal fold to 50% (of the original amplitude), see Figure 4 and Supplementary Video 2. Subsequently, the asymmetric motion reduces the corresponding maxima of the GAWs to 75% compared to the symmetric cases.

#### 2.1.4. Boundary Conditions

At all walls of the *simVoice* model, no-slip no-injection boundary conditions were applied. The walls of the moving vocal folds were defined as moving wall boundaries. For all simulation cases, the mean pressure of the subglottal inlet boundary is *P*_*inlet*_ = 775*Pa* that is in the physiologic range of human lunge pressures during normal phonation (Titze, 2000). The mean pressure at the outlet, which represents the mouth, is *P*_*outlet*_ = 0*Pa*. The kinematic viscosity of air was specified as $\nu =1.5666\cdot 10^{-5}\frac{m^{2}}{s}$ and the density of air constant at $\rho =1.18415\frac{kg}{m^{3}}$ as the Mach number is *Ma* < 0.3 (Kniesburges et al., 2011).

#### 2.1.5. Numerical Methods

The numerical setup is identical to the previous studies (Sadeghi et al., 2018, 2019a,b). To perform the simulations of *simVoice*, we use the software package STAR-CCM+ (Siemens, PLM Software, Plano, TX, USA) with a finite-volume cell-centered non-staggered grid. For modeling the turbulence, Large Eddy Simulations (LES) in combination with a Wall-Adapting Local Eddy-Viscosity (WALE) subgrid-scale model (Nicoud and Ducros, 1999) were carried out. The convective and diffusive terms of the Navier-Stokes equations were discretized with a central difference scheme with second-order accuracy. Subsequently, the pressure-correction PISO algorithm (Pressure-Implicit with Splitting Operators) solves the pressure-velocity linked equations non iteratively. Finally, an Algebraic Multigrid (AMG) method with a Gauss-Seidel relaxation scheme was applied to solve the final linear system of equations.

#### 2.1.6. Mesh Generation

**Figure 5:**
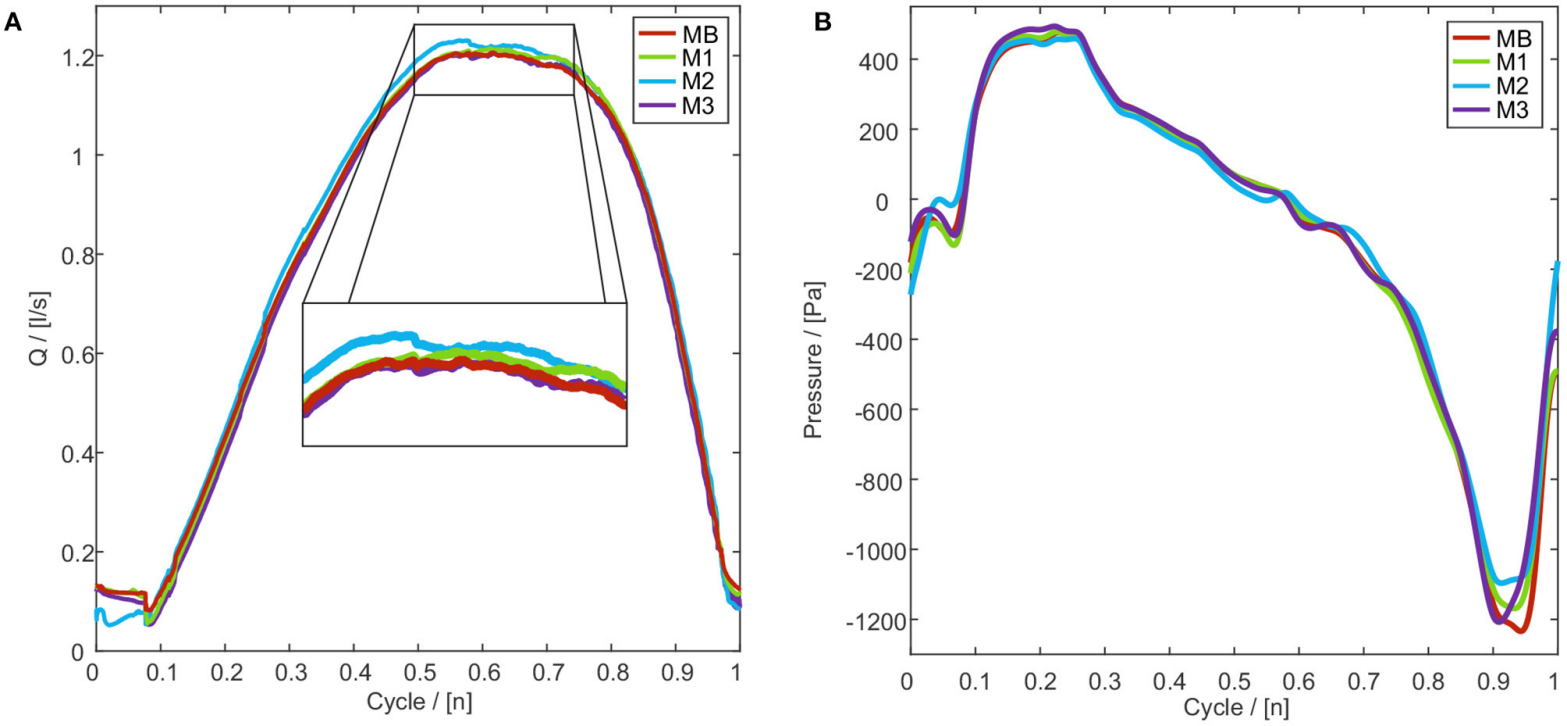
**(A)** Volume flow rate through the glottis for one oscillation cycle for different mesh resolutions MB-M3. **(B)** Instantaneous pressure evolution for mesh resolutions MB-M3 for one oscillation cycle at point P1, see Figure 2. The pressure evolutions were smoothed by a low-pass filter (Butterworth), with a cut-off frequency of 2,000 Hz, to reduce the numerical noise.

The mesh consists of hexahedral cells and is based on the mesh presented by Sadeghi et al. (2019b). For the mesh independence study, GC1 and a symmetric vocal fold motion was conducted. Starting with the base mesh (MB) with 2.9 million cells, three more meshes (M1-M3) with a decreasing number of cells were generated, see Supplementary Table 1. The limit for the mesh coarsening was set by the Taylor micro-scale λ_*T*_ = 0.085*mm* according to Mihaescu et al. (2010). Figure 5A) shows the flow rate for one oscillation cycle. M1-M3 produced a similar trend and the mean relative deviation to MB ranges between −1.3% and +2.6%, see Supplementary Table 1. Whereas M3 shows the best accordance with MB in the cycle range 1.4 to 1.8, M1 and M2 deviate from the trend of MB. Figure 5B shows an instantaneous pressure evolution at point P1 with a good agreement of meshes M1-M3 in comparison with mesh MB. Small deviations at the beginning and the end of the cycle are visible, which are the result of different instantaneous turbulent fluctuations at point P1 (Sadeghi et al., 2019b), see Figure 5B. Summarizing, M3 with the lowest number of cells shows good agreement with the base mesh MB. The resulting mesh M3 is assembled of 1.3 million cells with a basic cell size of 0.68 mm, see Supplementary Table 1.

The near-wall flow is modeled by the all-y+ model of Star-CCM+ that can handle fine and coarse meshes (Reichardt, 1951). The first cell layers on the vocal fold walls have a *y*+ = 1. The time step size is set to 1.36·10^−6^*s*, and the corresponding mean CFL number is 3.5 that is appropriate for implicit solvers (Anderson, 1995; Hirsch, 2007). *simVoice* uses the overset mesh approach of STAR-CCM+ to realize the vocal fold motion. This chimera method combines a fixed Eulerian background mesh with an Arbitrary Lagrangian-Eulerian (ALE) overlapping mesh (Hadzic, 2005). In *simVoice*, the mesh around both vocal folds represents the overlapping or overset mesh. Consequently, the total number of cells changes over time and depends on the GC type and the distance between the vocal folds during the oscillation.

### 2.2. *simVoice*—CAA Model

#### 2.2.1. Geometry Dimensions

The acoustic model of *simVoice* has been introduced by Schoder et al. (2020). According to the hybrid aeroacoustic approach, the acoustic domain captures the CFD domain assembled by the larynx and the vocal tract, where the acoustic sources occur. This region is coupled to a propagation domain in which the microphone points Mic1 and Mic2 are located, see Figure 2B. These points are positioned on the centerline of the vocal tract at a distance of 5 cm and 8 cm from the vocal tract exit (mouth). Additionally, perfectly matched layers (PML) surround the propagation domain to ensure free field radiation (Kaltenbacher, 2015). Owing to the plane wave approximation, we use an absorbing boundary condition (ABC) at the inlet that requires less computing power compared to PML (Kaltenbacher, 2015). Furthermore, all solid walls are modeled as acoustically hard.

To preserve mesh flexibility and element quality, the acoustic computation grid is composed of two conforming meshes linked via a non-conforming Nitsche-type mortaring interface. The mesh of the larynx and the vocal tract was generated for each GC type separately, representing the geometry of the maximum VF opening. It consists of tetrahedral finite elements with a maximum cell size of 5.7 mm. In contrast, the mesh in the propagation domain is the same for all GC types and has hexahedral elements with a cell size of about 10.9 mm.

#### 2.2.2. Numerical Methods

The aeroacoustic sound generation and acoustic wave propagation is described by the perturbed convective wave equation (PCWE) (Kaltenbacher et al., 2016), which is solved via the finite element solver CFS++ (Schoder et al., 2020). To compute the acoustic source term for the PCWE, the incompressible pressure field from the CFD is transferred onto the CAA mesh by a conservative interpolation scheme based on a cut cell algorithm (Schoder et al., 2019, 2020). The acoustic source term is then computed on the CAA grid as the partial time derivative of the incompressible pressure. We modeled a one-way coupling from the flow to the acoustic sources which was found to be valid for normal voice production (Schoder et al., 2020). A back-coupling effect from the acoustics to the flow field was not considered.

### 2.3. *simVoice*—Data Acquisition and Analysis

A total of 20 oscillation cycles of the vocal folds were simulated. In a first step, the *simVoice* CFD simulations were executed for 10 oscillation cycles to produce a fully developed flow field. After these 10 initializing oscillations, another 10 oscillation cycles were simulated to provide valid data for the analysis. As shown by Supplementary Figure 1 the model has achieved repeatable periodic oscillations with the flow field fully converged. The mean cyclic pressure at P1 fluctuates in the range of −7.1 and 9.1% and for P2 in the range of −9.1 and 6.5%, see Supplementary Figure 1A). These small fluctuations highly depend on the turbulent characteristic and the small cycle-to-cycle changes of the fluid flow in the supraglottal region (Kniesburges et al., 2016). The mean volume flow $\bar{Q}$ of the 10 initial oscillations is nearly constant and fluctuates in the range of −0.4 and 1.2%, see Supplementary Figure 1B). For the analysis, the complete 3D pressure and velocity fields were exported at every 10th time-step. These flow field data are then imported into CFS++ to determine the acoustic sources and to run the simulation of sound propagation. Finally, the acoustic signals at the two microphone positions were used. The sound pressure level (SPL) was calculated at a reference sound pressure of *p*_0_ = 20μ*Pa* using a Matlab (Mathworks, USA) routine. Therefore, the acoustic potential of Mic.2, see Figure 2B, was extrapolated to a distance of 20 cm far from the vocal tract outlet to match the distance of *ex vivo* studies (Birk et al., 2016, 2017b). The Vocal Efficiency (VE) is calculated as proposed by Riede et al. (2019) and Titze (1992):

(2) $$\begin{array}{r} \text{VE}=\frac{P_{\text{r}}}{P_{\text{a}}}=\frac{4\cdot \pi \cdot R^{2}\cdot 10^{\frac{SPL-120}{10}}}{P_{\text{sub}}\cdot \bar{Q}} \end{array}$$

where *P*_r_ is the radiated acoustic power, *P*_a_ is the aerodynamic power, *R* is the distance of the microphone to the opening of the vocal tract, *P*_sub_ is the subglottal pressure, and $\bar{Q}$ is the mean volume flow through the glottis. Additionally, the computed acoustic pressures were analyzed by the in-house Glottis Analysis Tool (GAT) for obtaining the Cepstral Peak Prominence (CPP) (Hillenbrand et al., 1994). The CPP is a spectra-based, well-established and objective measure to judge for perceived breathiness or vocal fatigue (Hillenbrand et al., 1994; Hillenbrand and Houde, 1996; Brinca et al., 2014; Samlan et al., 2014; Samlan and Story, 2017; Patel et al., 2018; Mahalingam et al., 2020; Murton et al., 2020) and has proven to be a more reliable measure of dysphonia than time-based measures (Heman-Ackah et al., 2003). The exact computation procedure is shown in Birk et al. (2016). The CFD data are evaluated concerning the volume flow through the glottis, the glottis resistance as proposed by van den Berg et al. (1957), and the energy transfer between the airflow and the vocal folds tissue. The energy transfer is defined by the work performed by the aerodynamic forces on the moving VFs according to Thomson et al. (2005).

## 3. Results

### 3.1. Aerodynamic Characteristics

#### 3.1.1. Volume Flow

**Figure 6:**
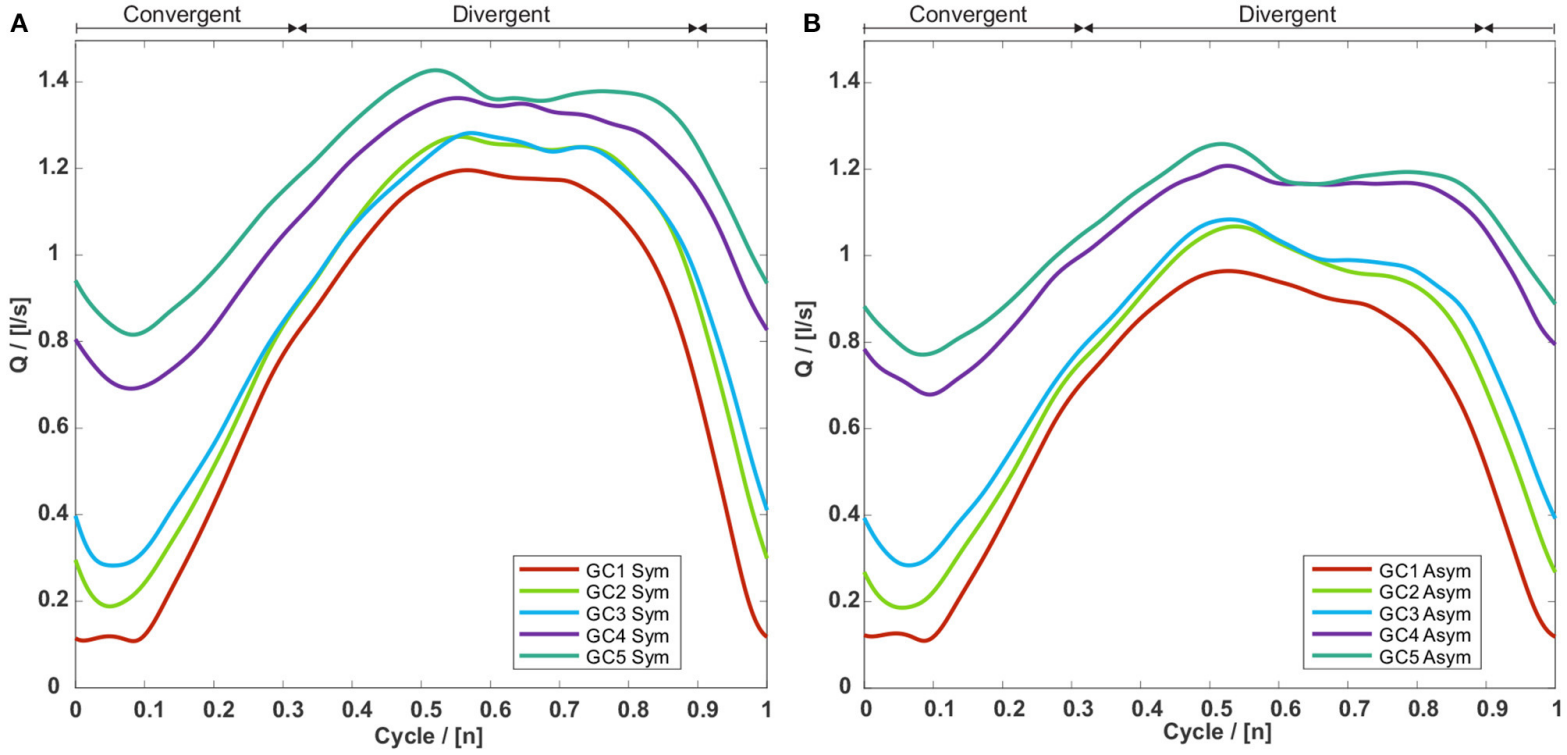
Volume flow through the glottis for the five GC types with **(A)** a symmetric and **(B)** an asymmetric vocal fold motion. For both motion types the volume flows are rising with an increasing glottal insufficiency, whereas the corresponding volume flows of the asymmetric motion are collectively smaller than those of the symmetric motion.

The minimum, maximum, and mean volume flow through the glottis consequently increases with an increasing glottal insufficiency from GC1 to GC5 for symmetric and asymmetric vocal fold motions as shown in Figure 6 and Table 2. The flow rate decrease comparing symmetric and asymmetric motion amounts between 9.0% (GC4) and 18.2% (GC1) as displayed in Table 2.

**Table 2:** Mean volume flow through the glottis $\bar{Q}$, the glottis resistance *R*_Glottis_, and the net energy *W*_net_ of all GC types.

**Parameter**	**GC1**	**GC2**	**GC3**	**GC4**	**GC5**
${\bar{Q}}^{\text{sym}}$ in $\left[\frac{l}{s}\right]$	0.77	0.88	0.91	1.11	1.20
${\bar{Q}}^{\text{asym}}$ in $\left[\frac{l}{s}\right]$	0.63	0.73	0.78	1.01	1.06
*rel*.*Dev*.	−18.2%	−17.0%	−14.3%	−9.0%	−11.7%
$R_{\text{Glottis}}^{\text{sym}}$ in $\left[\frac{Pa\cdot s}{m^{3}}\right]$	1168.9	1044.3	1003.2	845.6	915.0
$R_{\text{Glottis}}^{\text{asym}}$ in $\left[\frac{Pa\cdot s}{m^{3}}\right]$	1397.3	1262.0	1183.5	902.4	1032.3
*rel*.*Dev*.	19.5%	20.8%	18.0%	6.7%	12.8%
$W_{\text{net}}^{\text{sym}}$ in [μ*J*]	165.4	167.1	148.7	73.1	79.1
$W_{\text{net}}^{\text{asym}}$ in [μ*J*]	114.8	113.1	105.2	54.1	65.2
*rel*.*Dev*.	−30.6%	−32.3%	-29.3%	−26.0%	−18.0%

*Relative deviation (rel.Dev.) refers to deviation of asymmetric to symmetric motion values. $\bar{Q}$ increases while R_Glottis_ and W_net_ decrease with increasing glottal insufficiency. However, in contrast to R_Glottis_, W_net_ decreases for asymmetric motion owing to the smaller total amplitude of the glottis oscillation*.

#### 3.1.2. Glottis Resistance

The flow resistance across the glottal duct *R*_Glottis_ (Kniesburges et al., 2017) decreases with an increasing glottal insufficiency. The reason for this decrease in *R*_Glottis_ is the rising flow rate $\bar{Q}$, while the *P*_sub_ remains constant. The direct comparison of *R*_Glottis_ between symmetric and asymmetric vocal fold motion yielded a larger resistance for the asymmetric motion because $\bar{Q}$ is reduced owing to the smaller glottal gap, see Table 2.

#### 3.1.3. Energy Transfer

As proposed by Sadeghi et al. (2019a), the total transferred work (*W*_net_) during one oscillation cycle is calculated, see Table 2. For both motion types, the total net work during an oscillation cycle is positive, being typical for vocal fold oscillations during phonation (Thomson et al., 2005; Luo et al., 2009). Furthermore, *W*_net_ decreases with an increasing glottal insufficiency. Table 2 shows that *W*_net_ decreases by 55.8% (symmetric) and 52.9% (asymmetric) from GC1 to GC5 whereas the maximum deviation comparing symmetric and asymmetric motion occurs for GC2 with 32.3%. However, in contrast to *R*_Glottis_, *W*_net_ decreases for asymmetric motion owing to the smaller total amplitude of the glottis oscillation. Overall, our data shows that a partially closed glottis (GC2 and GC3) in combination with an asymmetric motion produces a higher *W*_net_ than a contact-free symmetric oscillation, see Table 2.

**Figure 7:**
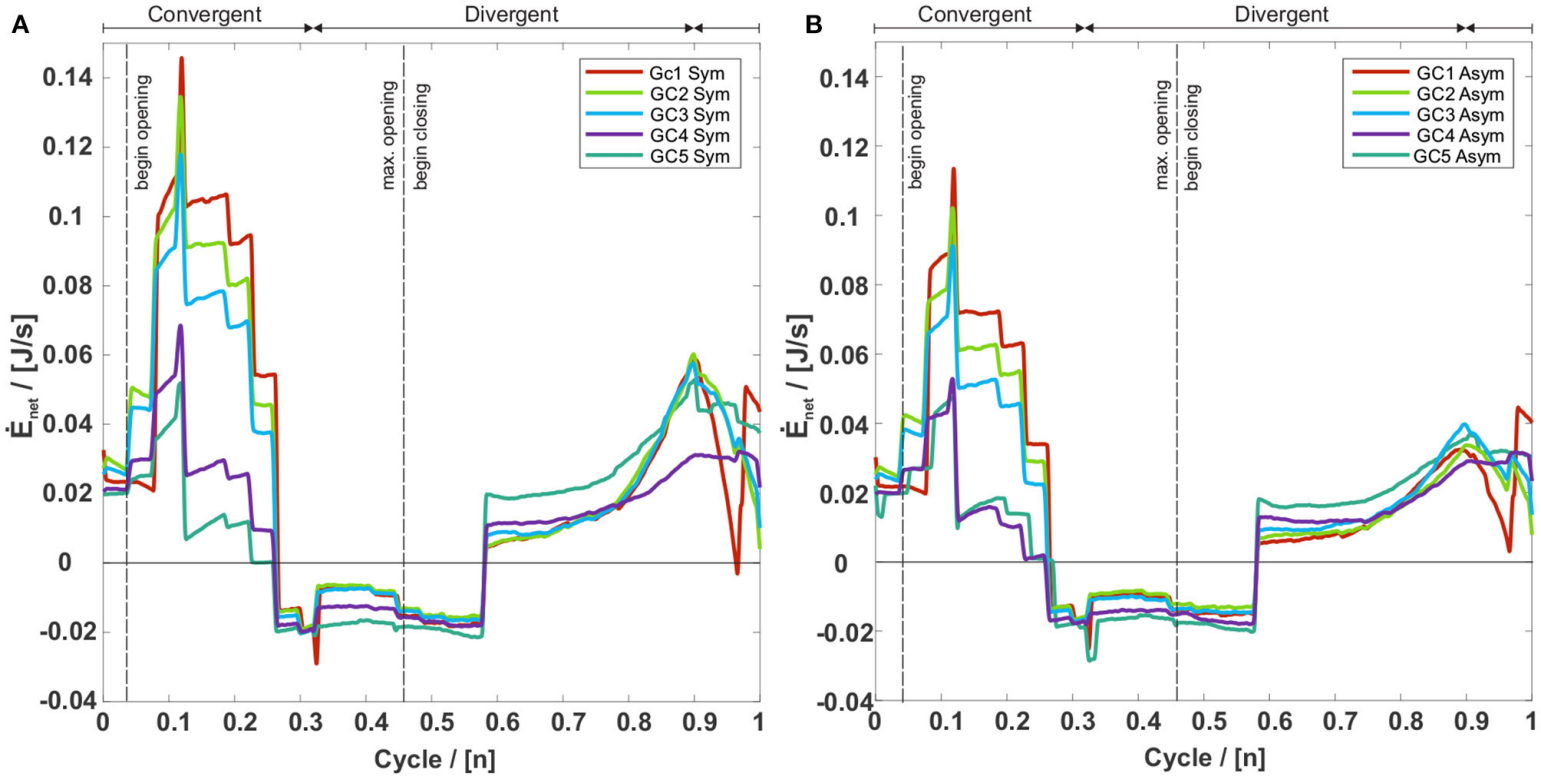
Net rate energy transfer (Ė_net_) of the five GC types with **(A)** a symmetric and **(B)** an asymmetric vocal fold motion. A positive Ė_net_ means an energy flux from the glottal flow toward the vocal folds and a negative Ė_net_ an energy flux from the vocal folds toward the airflow. For both motion types Ė_net_ is positive at the beginning and the end of the oscillation cycle. In these intervals Ė_net_ decreases with an increasing glottal insufficiency, whereas the corresponding values of the asymmetric motion are collectively smaller than those of the symmetric motion.

According to Sadeghi et al. (2019a), the time derivative of the work constitutes the net energy transfer rate Ė_net_ between fluid and tissue. It is shown in Figure 7 for both symmetric and asymmetric vocal fold motions. A positive Ė_net_ corresponds to an energy flux from the laryngeal flow into the tissue, i.e., the flow deforms the vocal folds (Sadeghi et al., 2019a). During the opening, until 0.25*T*, Ė_net_ is positive, which indicates the tissue deformation by the laryngeal flow. Between 0.25*T* to 0.58*T*, the glottis width reaches its maximum, producing a negative Ė_net_, resulting from the tissue's resistance to deform further (Sadeghi et al., 2019a). After the flow is fully accelerated, the aerodynamic pressure between the vocal folds is minimal, which initiates the glottis's closing motion. The VFs move toward each other, starting at 0.58*T*, and again a positive Ė_net_ arises. Although the motion of the vocal folds is prescribed in this model, Luo et al. (2009) show a similar energy transfer rate during a cycle of flow-induced VF oscillations. For clarity, we want to mention that the discrete changes in the energy transfer plots occur due to the frame rate of 4,000 fps of the camera, which was used to record the oscillations of the synthetic vocal folds (Kniesburges et al., 2013). Based on this recording the motion of the vocal folds was modeled without further smoothing and therefore discrete changes in the velocity subsequently occur at multiples of 0.25 ms. Figure 7 further shows that the positive Ė_net_ during the opening and closing phases decreases with an increasing glottal insufficiency. Furthermore, in the opening and closing phase, Ė_net_ is lower for the asymmetric motion, whereas it is equal for both motion types during the phase of significant tissue resistance (0.25*T*- 0.58*T*).

#### 3.1.4. Flow Field Structure

**Figure 8:**
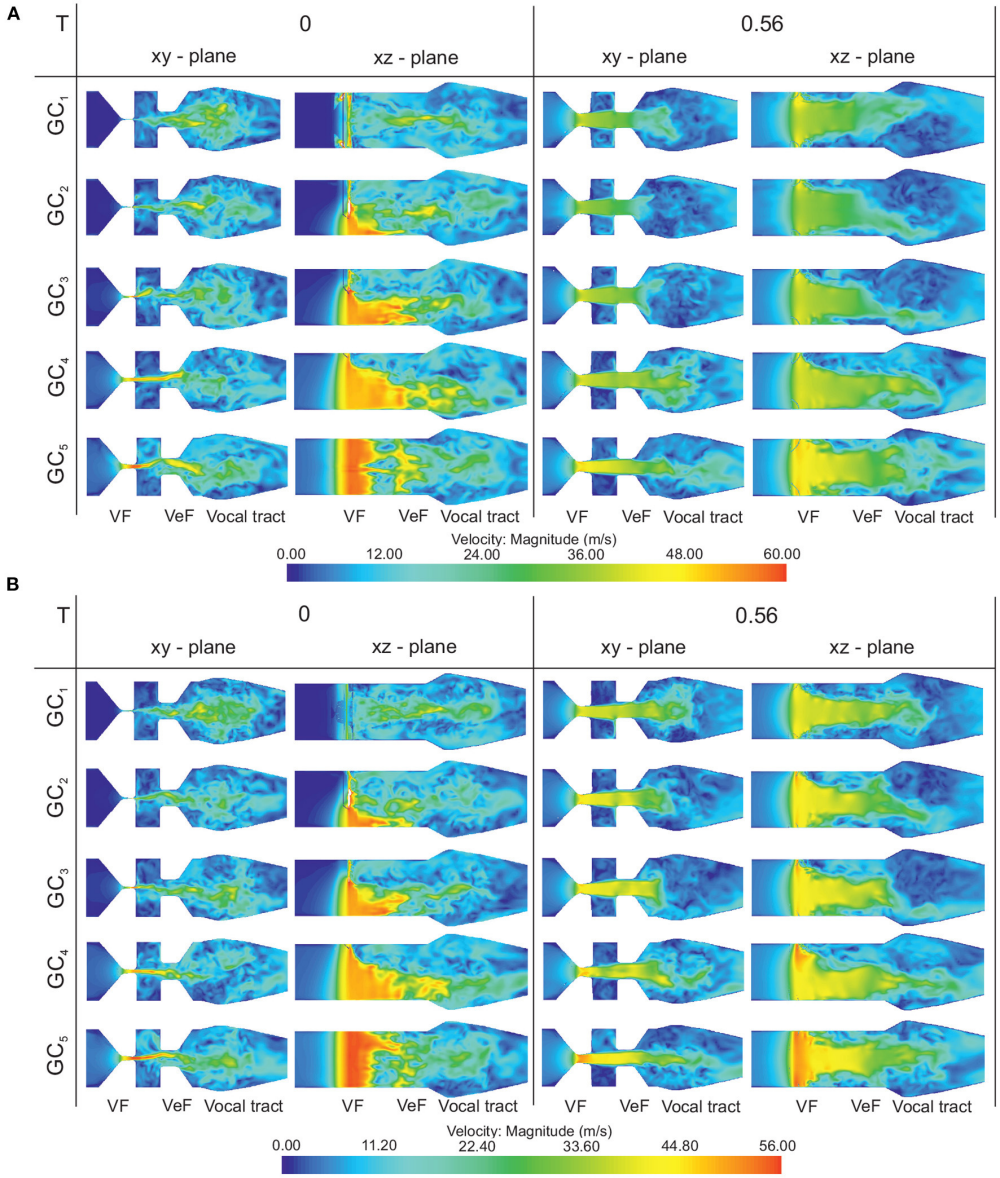
**(A)** Symmetric vocal fold motion: velocity magnitude in the midcoronal (xy-plane) and the sagittal (xz-plane) plane for the five GC types at two instances (*t*_1_ = 0 and *t*_2_ = 0.56*T*) of an oscillation cycle. While GC1 fully interrupts the glottal jet at the end of the cycle, GC2 and GC3 only partly, and GC4 and GC5 do not interrupt the laryngeal fluid flow. **(B)** Asymmetric vocal fold motion: Velocity magnitude in the midcoronal (xy-plane) and the sagittal (xz-plane) plane for the five GC types at two instances (*t*_1_ = 0 and *t*_2_ = 0.56*T*) of an oscillation cycle. The upper vocal fold moves with the 50% amplitude and the glottal jet impinges mainly the lower VeF and subsequently, just a vortex in the lower ventricle occurs.

Figure 8 shows the supraglottal flow field at two time instances (*t*_1_ = 0 and *t*_2_ = 0.56*T*) during the oscillation cycle for the symmetric and the asymmetric vocal fold motion. For all GC types, a long jet expands into the supraglottal region. While GC1 fully interrupts this glottal jet at the end of the cycle, GC2 and GC3 only partly interrupt the laryngeal fluid flow at the anterior section of the glottis. For GC4 and GC5, the vocal folds remain open along the entire glottis length during the oscillation cycle, see Supplementary Videos 3, 4. This absent interruption of the glottal jet is often related to an aspirated voice signal characterized by lower tonal sound components (Fritzen et al., 1986; Bhatt and Verma, 2014; Kniesburges et al., 2020). As reported by Sadeghi et al. (2018), the VeFs have a stabilizing influence on the glottal jet. Therefore, no jet deflection in the medial-lateral directions (Figure 8 in the xy-plane) can be observed, see Supplementary Videos 5, 6. However, the glottal opening shape has a strong influence on the posterior-anterior jet shape (Figure 8 in the xz-plane), see also Supplementary Videos 7, 8. As similarly reported by Zörner et al. (2016), triangular glottal orifices deflect the jet toward the larger glottal opening that occurs for GC2 and GC3 at the posterior end of the glottis.

For the symmetric vocal fold motion, the glottal jet impinges both VeF during the oscillation cycle and vortices arise in both ventricles. For the asymmetric case, the glottal jet impinges mainly the lower VeF and subsequently, just a vortex in the lower ventricle occurs, see Figure 8B for *t*_2_ = 0.56*T* in the xy-plane. Furthermore, the maximum glottal velocity is higher for the symmetric vocal fold motion than for the asymmetric vocal fold motion due to the larger flow rate in the symmetric cases, see color bars in Figure 8.

### 3.2. Quality of Acoustic Voice Signal

#### 3.2.1. Spectral Analysis and Formant Frequencies

**Figure 9:**
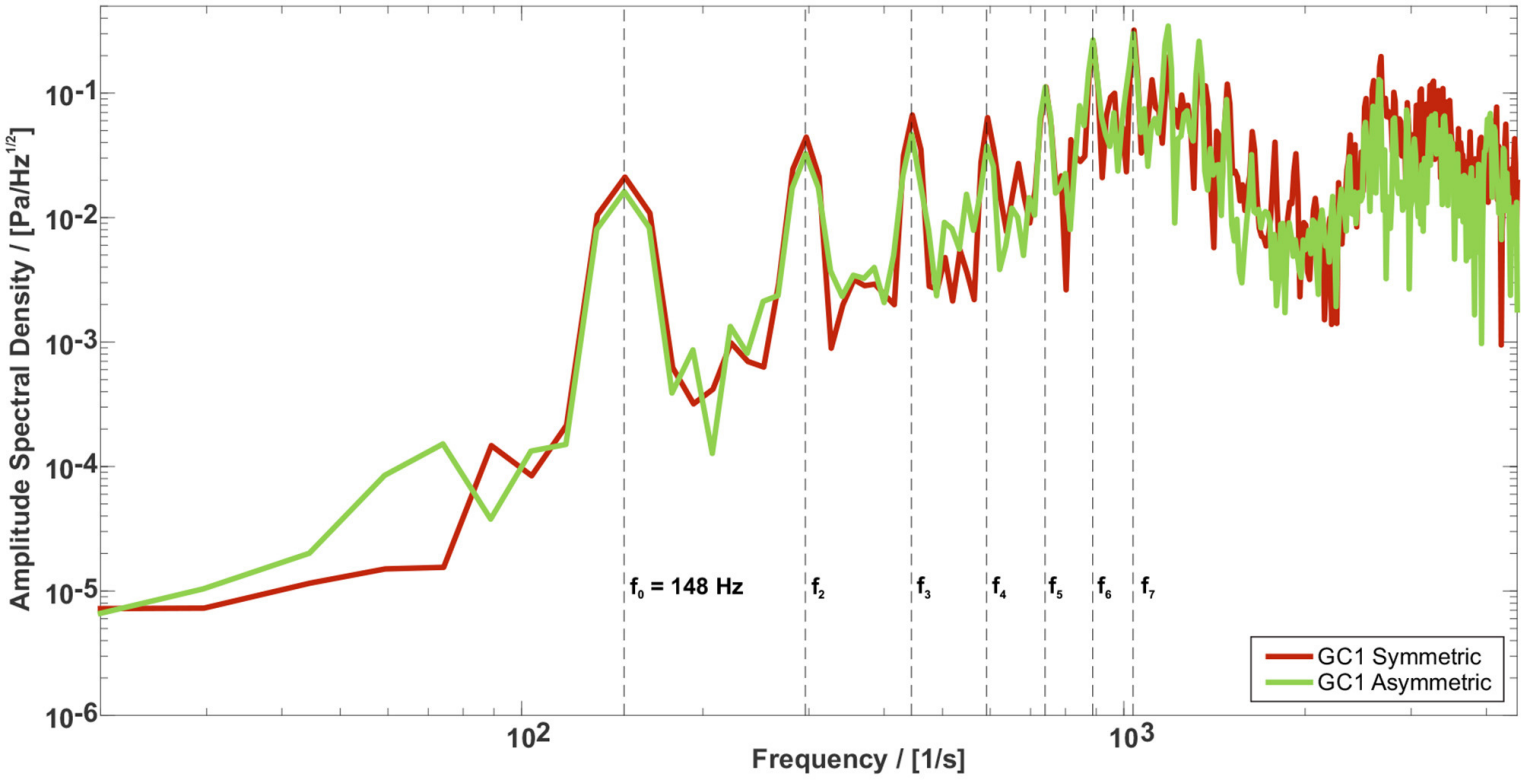
Amplitude Spectral Density (ASD) for the GC1 type for the symmetric and asymmetric vocal fold motion. The spectra of both motions show similar slope and only slight deviations in the amplitudes at the fundamental frequency, whereas more significant differences occur at higher harmonics.

Figure 9 shows the amplitude spectral density (ASD) of the sound signals for GC1 and both symmetric and asymmetric vocal folds motions measured at the Mic.1 position, see Figure 2B. Both spectra exhibit the main peak at the oscillation frequency of the vocal folds *f*_0_ = 148*Hz*, followed by their harmonics. Comparing the spectra of all GC types shows similar slope and only slight deviations in the amplitudes at the fundamental frequency, whereas more significant differences at the higher harmonics occur, see Supplementary Figures 2, 3. Regarding the motion type of the vocal folds, the harmonic tones are more pronounced for the symmetric vocal fold motion, especially in the frequency range between 1, 000 and 2, 000*Hz*. This variance in the acoustic spectra of the radiated sound was also found by Zörner et al. (2016) although the velocity fields of the five GC types are considerably different.

**Figure 10:**
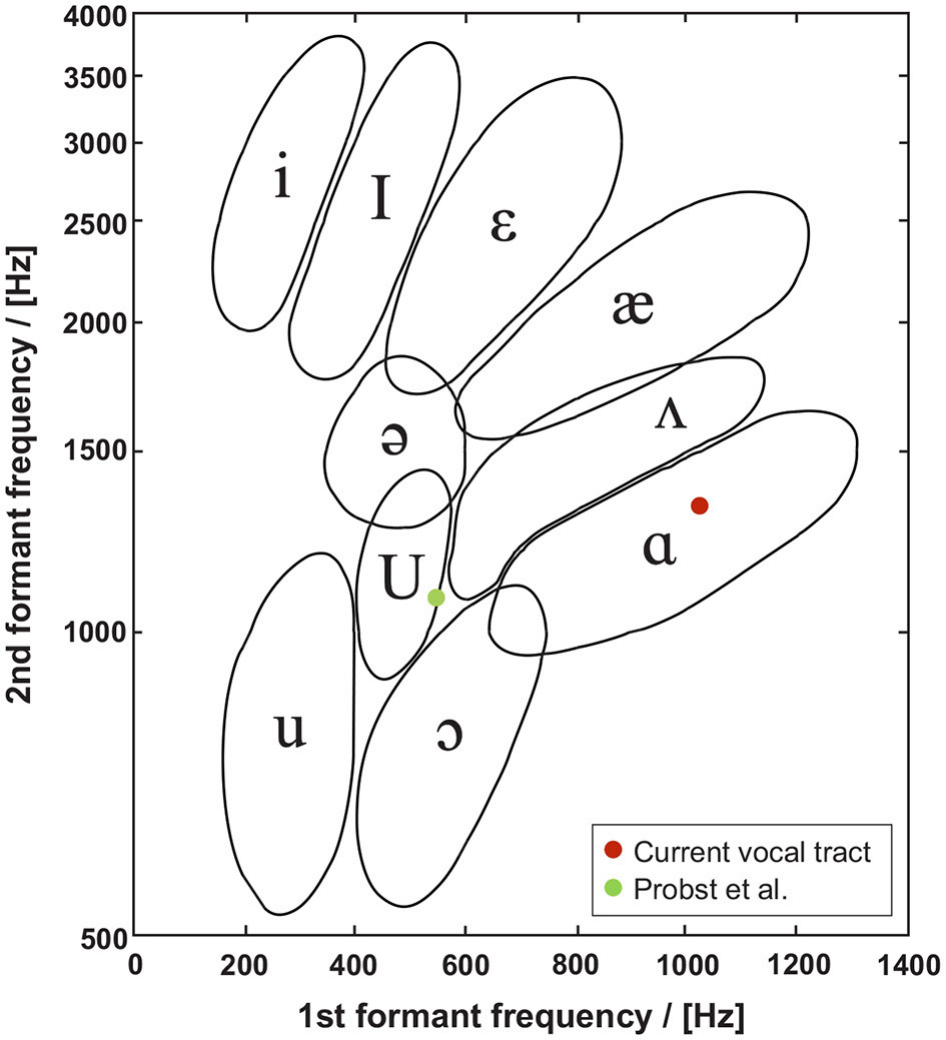
Formant chart as proposed by Peterson and Barney (1952), shows the formant frequencies of the first two formants found in this study and that of Probst et al. (2019). In contrast to Probst et al. (2019), *F*1 = 1, 020*Hz* and *F*2 = 1, 350*Hz* simulated by *simVoice* are well-positioned within the region of the /a/ vowel.

A modal analysis of the vocal tract shows that the first two formants *F*_1_ = 1, 020*Hz* and *F*_2_ = 1, 350*Hz*, see transfer function of /a/ vocal tract in Supplementary Figure S4, are well-positioned within the region of the /a/ vowel of the formant chart of Peterson and Barney (1952), shown in Figure 10.

#### 3.2.2. Sound Pressure Level (SPL) and Vocal Efficiency (VE)

**Figure 11:**
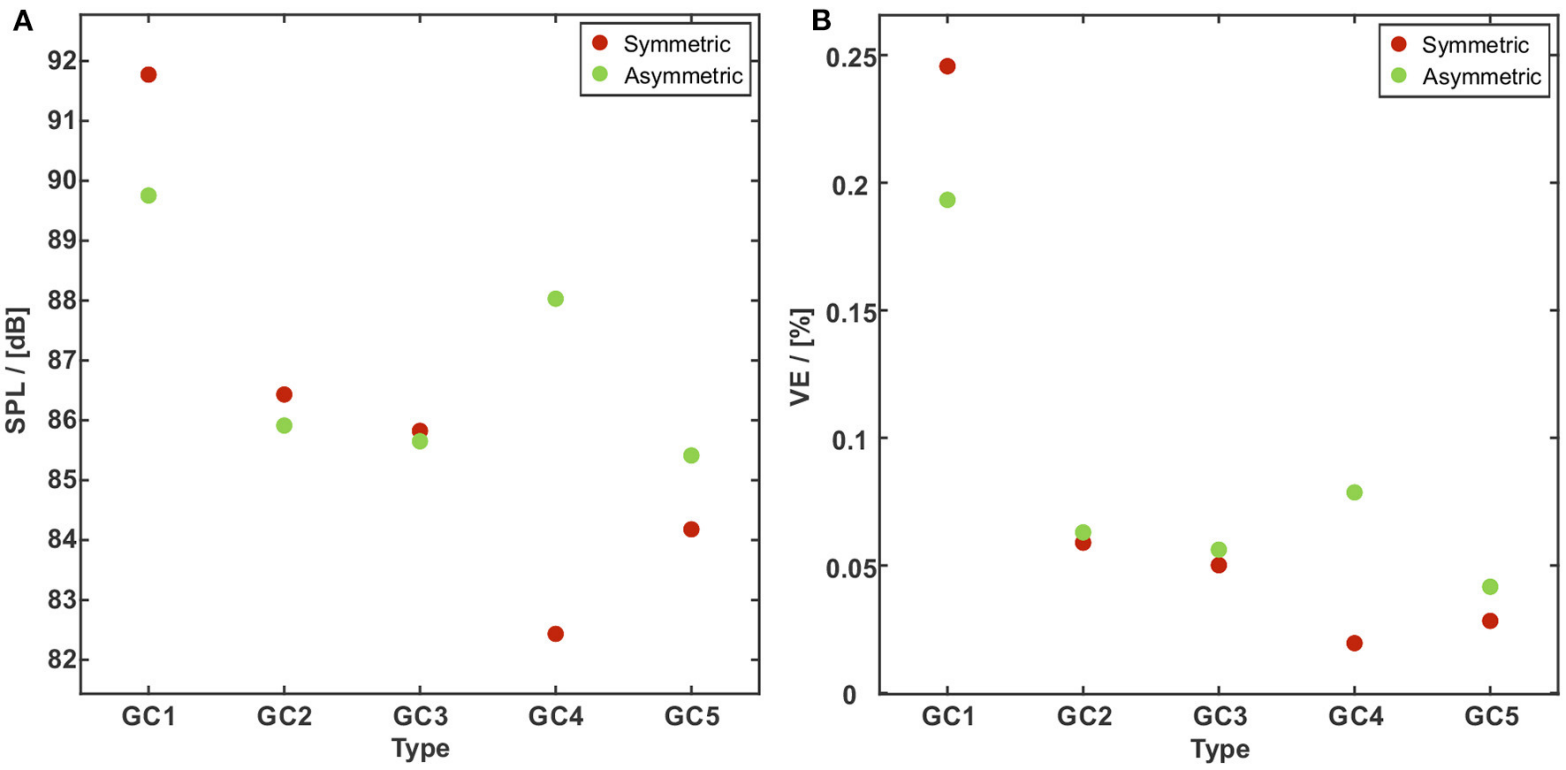
SPL **(A)** and VE **(B)** vs. the GC types with a symmetric (red dots) and an asymmetric (green dots) vocal fold motion. The SPL and the VE significantly decrease with an increasing glottal insufficiency. The comparison between both motion types shows significant differences for GC1 and GC4 and only minor differences for GC2, GC3, andGC5.

Figure 11A presents the SPL for all GC types. SPL significantly decreases with an increasing glottal insufficiency: For the symmetric motion type from 91.8 dB for GC1 to 82.4 and 84.2 dB for GC4 and GC5 representing a loss of 10.2 and 8.3%. For the asymmetric motion type, a decrease of about 4.5% for GC2 and GC3, 1.9% for GC4, and 4.9% for GC5, was found compared to SPL = 89.8 dB for GC1. The comparison between both motion types shows only minor differences. A maximum deviation of 6.4% for a higher SPL at the asymmetric motion occurs at GC4. Figure 11B shows the VE of all GC types. As mentioned before, the VE is the ratio of radiated acoustic power to aerodynamic power, see Equation (1). According to the SPL, the VE decreases for both vocal fold motion types (symmetric vs. asymmetric) and an increasing degree of glottal insufficiency (GC1 to GC5). The VE decreases from VE = 0.25% for GC1 to VE = 0.03% for GC5 for the symmetric motion and for the asymmetric motion, VE decreases less, from VE = 0.19% (GC1) to VE = 0.04% (GC5).

#### 3.2.3. Cepstral Peak Prominence (CPP)

**Figure 12:**
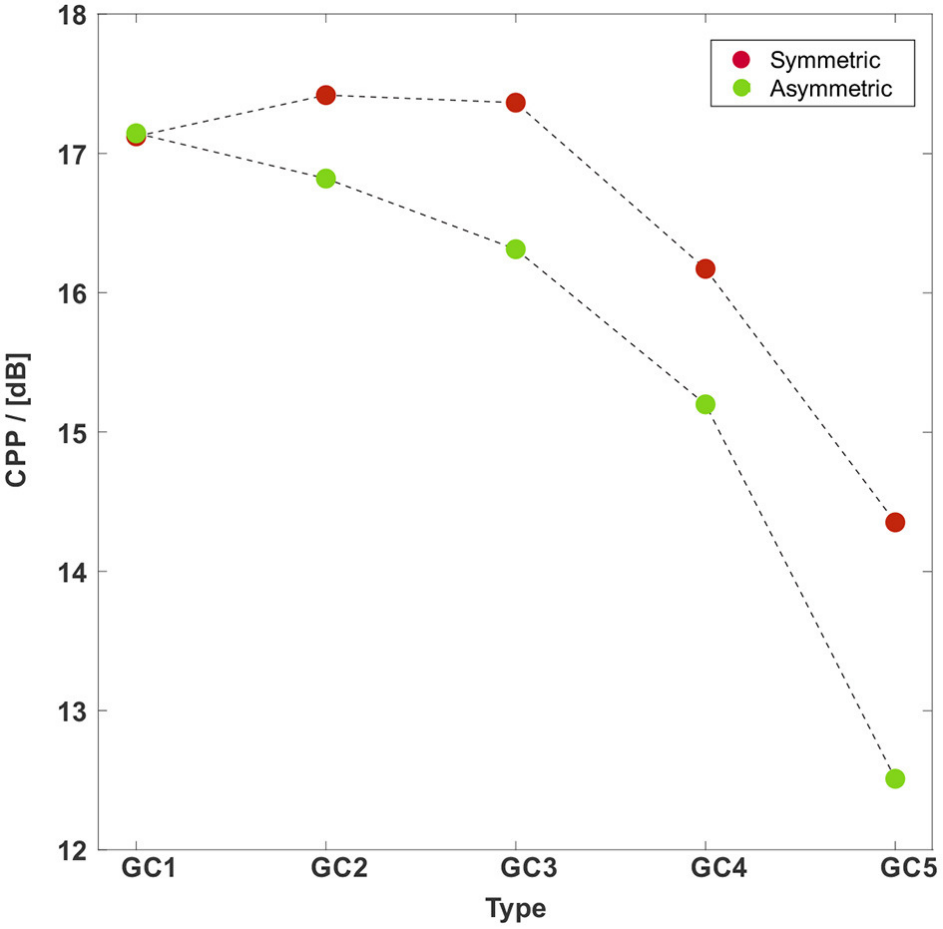
CPP vs. the GC types with a symmetric (red points) and an asymmetric (green points) vocal fold motion. The CPP for the symmetric vocal fold motion almost remains at the same level for GC1 to GC3 followed by a decrease. The CPP for the asymmetric vocal fold motion decreases for an increasing glottal insufficiency. The CPP for the asymmetric motion is collectively smaller than those for the symmetric motion.

The CPP is widely used as a quantitative measure for the periodicity of a signal and thereby has proven to be a reliable indicator for the strength of tonal components and therewith the quality of the human voice (Hillenbrand et al., 1994; Hillenbrand and Houde, 1996; Birk et al., 2017b). It is shown in Figure 12 for both motion types. The CPP for the symmetric vocal fold motion starts at 17.1 for GC1 and increases to 17.4 for GC2 and GC3. Afterwards, the CPP decreases to 16.2 dB for GC4 and further to 14.4 dB for GC5. For the asymmetric vocal fold motion the CPP decreases for an increasing glottal insufficiency from 17.1 dB for GC1 to 12.5 dB for GC5.

## 4. Discussion

### 4.1. Aerodynamic Characteristics

Our results of the volume flow through the glottis agree with the study by Zañartu et al. (2014) who reported an airflow rise with an increasing posterior gap. As the maximum glottal gap area of an asymmetric type is smaller than its symmetric equivalent, the mean volume flow $\bar{Q}$ is subsequently decreased, see Table 2. The left-right asymmetry does not only affect the maximum glottal area as reported by Pickup and Thomson (2009) but also significantly reduces the volume flow through the glottis for a constant inlet pressure in both motion types.

In phonation, the goal is to increase the energy transfer between the glottal airflow and the VFs as a beneficial mechanism to induce the VF oscillation. Kniesburges et al. (2017) interpreted the flow resistance as a measure of energy transfer from the glottal flow to the VFs. Furthermore, Birk et al. (2017b) reported that the energy transfer from the glottal airstream to the vocal folds, as indicated by the glottal resistance, is strongly dependent on glottal insufficiency. In this context, a complete glottis closure during the VFs oscillation produces a large flow resistance *R*_Glottis_ and in addition a large energy transfer between flow and tissue. Additionally, our data support the findings of Döllinger et al. (2018) which showed that a partially closed glottis (GC2 and GC3) in combination with an asymmetric motion may be still better than a contact-free symmetric oscillation.

In all GC cases, the interaction of the jet with the flow structures in the immediate supraglottal area causes deflection of the tail of the glottal jet. Zhang and Mongeau (2006) reported that this interaction leads to pronounced shear layers between the jet and the resting fluid with large velocity fluctuations.

### 4.2. Quality of Acoustic Signal

As described above, the vocal tract model is the smoothed version of the staged model developed by Probst et al. (2019). They reported formant frequencies of *F*_1_ = 550*Hz* and *F*_2_ = 1, 080*Hz*, being lower than the formants found here. We assume the shift of the first two formants in this study to higher values is due to the vocal tract smoothing. As reported by Jiang et al. (2017) the location of the formants and a resulting shift significantly depends on the area variation along the tract. Probst et al. (2019) and Jiang et al. (2017) found lower frequencies for the first two formants, but Jiang et al. (2017) used a vocal tract, mimicking a neutral vowel /schwa/ superimposed onto a realistic airway centerline from *in vivo* MRI measurements. Comparing the third formant *F*_3_ of our model with that of Probst et al. (2019) shows a good agreement.

Moreover, the results of SPL show good qualitative agreement with those reported by Thornton et al. (2019) and Döllinger et al. (2018), see Table 3. They executed *ex vivo* experiments with rabbit larynxes and three different glottal insufficiency grades (complete glottal closure, partial glottal closure, no contact of vocal folds). They measured the SPL at a distance of 20 cm from the glottis. Furthermore, our SPL is higher than the *in vivo* measurements of Södersten et al. (1995) because the microphone in our model is located 30 cm closer to the vocal folds, nevertheless our SPL values are in the human range (Gramming et al., 1988). Our results show that an increasing posterior gap and glottal insufficiency may reduce the SPL as reported by Zañartu et al. (2014).

**Table 3:** SPL in [dB] of Döllinger et al. (2018) and Thornton et al. (2019).

**SPL in [dB]**	**GC1**	**GC2/GC3**	**GC4**
	**closed**	**partially closed**	**no contact**
Döllinger et al., 2018	79.1 ± 6.4	76.1 ± 7.1	69.4 ± 7.5
Thornton et al., 2019	76.7 ± 6.5	76.0 ± 7.6	59.4 ± 7.5

*Even though our SPL values are higher they show good qualitative agreement with the trends reported in this study*.

Tanaka and Gould (1985) found a low VE with a large glottal gap and a high flow rate. Due to the dependency of the radiated acoustic power from the mouth opening and therefore from the vowels (Titze et al., 2016), our results may be just valid for a vowel /a/. Although the basic trend of the VE for the asymmetric motion coincides with that for symmetric motion, the VE is mostly larger (GC2 to GC5) compared to the symmetric motion and is just lower for GC1. Thus, our results agree for GC1 with the study by Oren et al. (2016), who reported a reduction of VE for asymmetric vocal fold motion (the study does not present the degree of glottal insufficiency). We could not identify a discrete effect that produces the outlier in SPL and subsequently in VE for GC4. We assume a cumulative effect that may occur mainly in the higher frequency range of the acoustic signal.

Both effects, an increasing insufficiency, and an asymmetric vocal fold motion potentially reduce the tonal components of the acoustic signal and the voice quality. The same observations have been made in *in vivo* studies executed by Samlan et al. (2014) and Chen et al. (2011). Furthermore, the qualitative trend of CPP was also found in *ex vivo* studies with human (Birk et al., 2017b) and rabbit larynges (Döllinger et al., 2018; Thornton et al., 2019), as shown in Table 4. The high CPP values for GC2 and GC3 for symmetrically oscillating VFs shows, that the acoustic signal is still tonal and physiological for small posterior gaps as often observed in physiological phonation of women and childs (Södersten and Lindestad, 1990; Södersten et al., 1995; Inwald et al., 2010; Patel et al., 2012; Kniesburges et al., 2020). Quantitatively, our CPP values are in the range of values reported by Döllinger et al. (2018) and Thornton et al. (2019).

**Table 4:** CPP in [dB] of Birk et al. (2017b), Döllinger et al. (2018), and Thornton et al. (2019).

**CPP in [dB]**	**GC1 closed**	**GC2 30% partially closed**	**GC3 60% partially closed**	**GC4 no contact**
Birk et al., 2017b	24.3 ± 5.82	21.8 ± 4.2	16.4 ± 2.82	15.7 ± 1.94
Döllinger et al., 2018	24.0 ± 4.8	22.8 ± 4.8		19.4 ± 4.9
Thornton et al., 2019	17.9 ± 4.3	15.8 ± 6.5		11.0 ± 3.4

*Quantitatively, our CPP values are in the range of values reported there*.

### 4.3. Limitations of the Study

The vocal fold vibration in this study is prescribed, neglecting the fluid-structure interaction (FSI), which is a common approach to increase the efficiency of the simulations.

## 5. Conclusion

Glottal insufficiency and asymmetric vocal fold oscillations have been investigated using our numerical aeroacoustic model *simVoice*. Aerodynamically, an increasing degree of glottal insufficiency leads to a decrease in flow resistance and a decrease in the energy transfer rate between flow and tissue. This means a reduction of the stimulation of the vocal fold oscillations and subsequently impairs the acoustic signal. Thus, CPP (Hillenbrand and Houde, 1996; Birk et al., 2017b; Döllinger et al., 2018; Thornton et al., 2019), SPL (Döllinger et al., 2018; Thornton et al., 2019), and VE (Tanaka and Gould, 1985) deteriorate for an increasing degree of glottal insufficiency.

All these findings correlate with symptoms of functional voice disorders such as breathiness, hoarseness, and an enhanced effort needed to phonate, commonly called air loss during phonation (Fritzen et al., 1986; Zhang, 2019). However, a glottis insufficiency can also occur in physiological phonation often observed in women and children who have a triangular-shaped opening located in the posterior glottis (Södersten and Lindestad, 1990; Södersten et al., 1995; Inwald et al., 2010; Patel et al., 2012; Kniesburges et al., 2020). Those persons have often a soft and quiet voice as reported by Fritzen et al. (1986) and Bhatt and Verma (2014).

In principle, the same trend of a deterioration for an increasing degree of glottal insufficiency for CPP, SPL and VE can be seen when comparing symmetric and asymmetric motion of the vocal folds: The energy transfer rate and the acoustic parameters decrease for asymmetric motion. However, this trend is not that distinct as for glottal insufficiency (Birk et al., 2017b). Therefore, a left-right asymmetry must not necessarily result in a salient reduction in voice quality, as similarly reported by Zhang et al. (2012).

From our results, we assume that a high degree of glottal insufficiency potentially displays the most severe symptom for a functional voice disorder, which has to be focused on during clinical treatment [e.g., medialization with hyaluronic acid-based materials or thyroplasty (type 1 thyroplasty)]. Thereby, the asymmetry of the motion of the vocal folds seems to have a reduced role in negatively impacting the voice quality compared to the glottal insufficiency. But both symptoms in combination will further reduce the quality of the sound signal.

Regarding the functionality of *simVoice*, the study shows: (1) *simVoice* can mimic simplified vibration characteristics and glottal geometries, (2) *simVoice* reveals separated and combined effects of aerodynamic and acoustic symptoms of a glottal insufficiency and an asymmetric vocal fold motion, and (3) a current walltime of 10 h/cycle is, with a prospective increase in computing power, very promising for a clinical approach.

Furthermore, CFD data in addition to experimental data are essential to develop, train and validate neural networks as done by Zhang (2020) and Zhang et al. (2020), which will further speed up the computing time of the phonation process and the implementing of numerical models in the clinical environment.

## Data Availability Statement

The original contributions presented in the study are included in the article/supplementary material, further inquiries can be directed to the corresponding author/s.

## Author Contributions

MD, MK, and SK conceived the study and contributed to data analysis and interpretation, supervision, and manuscript writing. SF conducted main writing and review editing. SF, BJ, SS, and PM conducted the CFD and CAA simulations, and contributed to data analysis, interpretation and manuscript writing. ME contributed to results interpretation, clinical input, review editing and provided the VT geometry. All authors contributed to the article and approved submitted version.

## Conflict of Interest

The authors declare that the research was conducted in the absence of any commercial or financial relationships that could be construed as a potential conflict of interest.
