# Supplementary material for: 3D-FV-FE Aeroacoustic Larynx Model for Investigation of Functional Based Voice Disorders
Source: Front Physiol. 2021 Mar 8;12:616985. doi: 10.3389/fphys.2021.616985 (PMC7982522; doi:10.3389/fphys.2021.616985)
Supplement: Supplementary file 9 [file Data_Sheet_1.PDF]

Supplementary Material

1 SUPPLEMENTARY DATA

1.1 Videos

Videos:

- V1: Symmetric vocal fold motion,
- V2: Asymmetric vocal fold motion,
- V3: Symmetric motion, isosurface velocity,
- V4: Asymmetric motion, isosurface velocity,
- V5: Symmetric motion, velocity in xy-plane,
- V6: Asymmetric motion, velocity in xy-plane,
- V7: Symmetric motion, velocity in xz-plane, and
- V8: Asymmetric motion, velocity in xz-plane.

2 SUPPLEMENTARY TABLES AND FIGURES

2.1 Figures

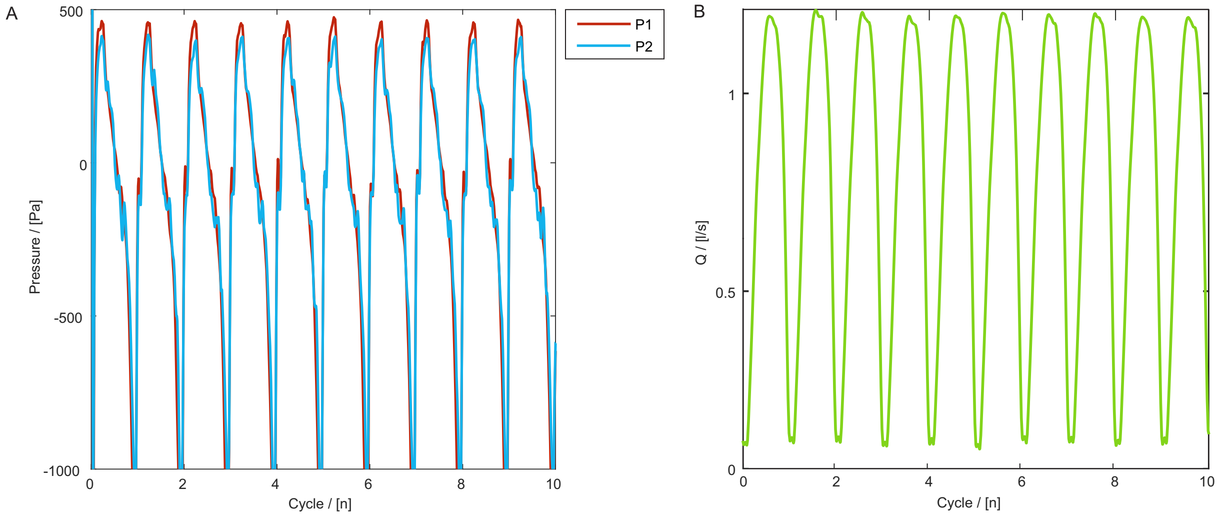

Figure S1. A) Instantaneous pressure evolutions for mesh M3 at point P1 and P2 (introduced in Fig. 2) for the ten oscillation cycles that were analyzed. The pressure evolutions were smoothed by a low-pass filter (Butterworth) wit a cut-off frequency of 2000 Hz) to reduce the numerical noise. B) Volume flow rate through the glottis for mesh M3 for the ten oscillation cycles that were analyzed.

2.2 Tables

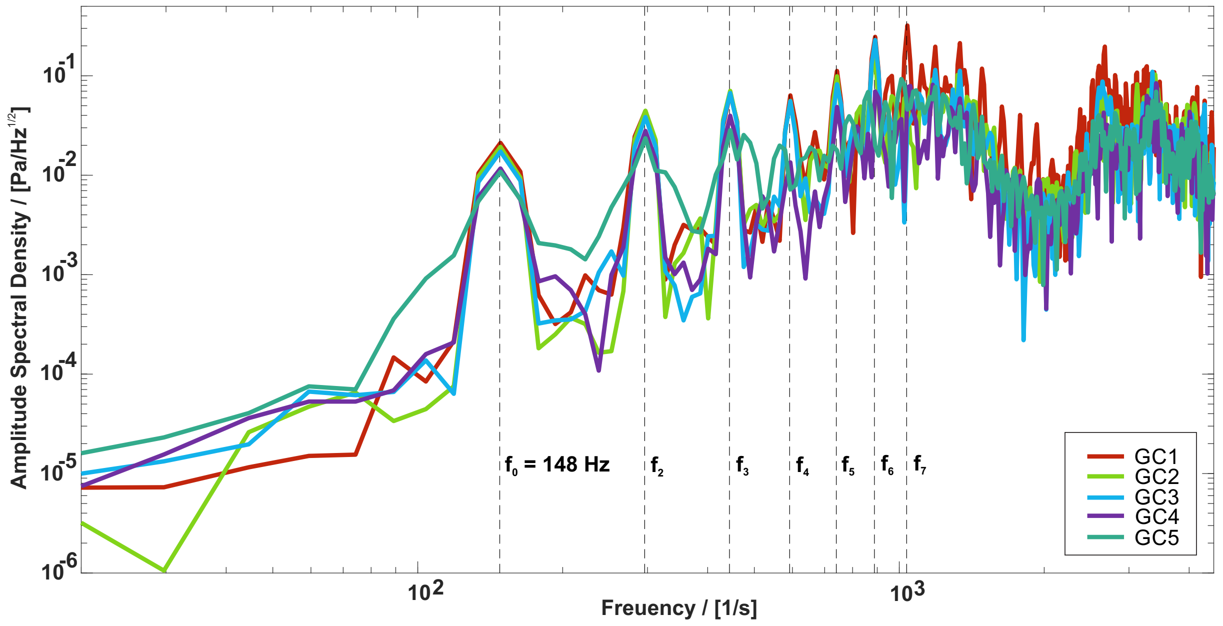

Figure S2. Amplitude Spectral Density (ASD) for the five GC type with symmetric vocal fold motion. The spectra of the five GC types show similar slops and only slight deviations in the amplitudes at the fundamental frequency, whereas more significant differences at the higher harmonics occur.

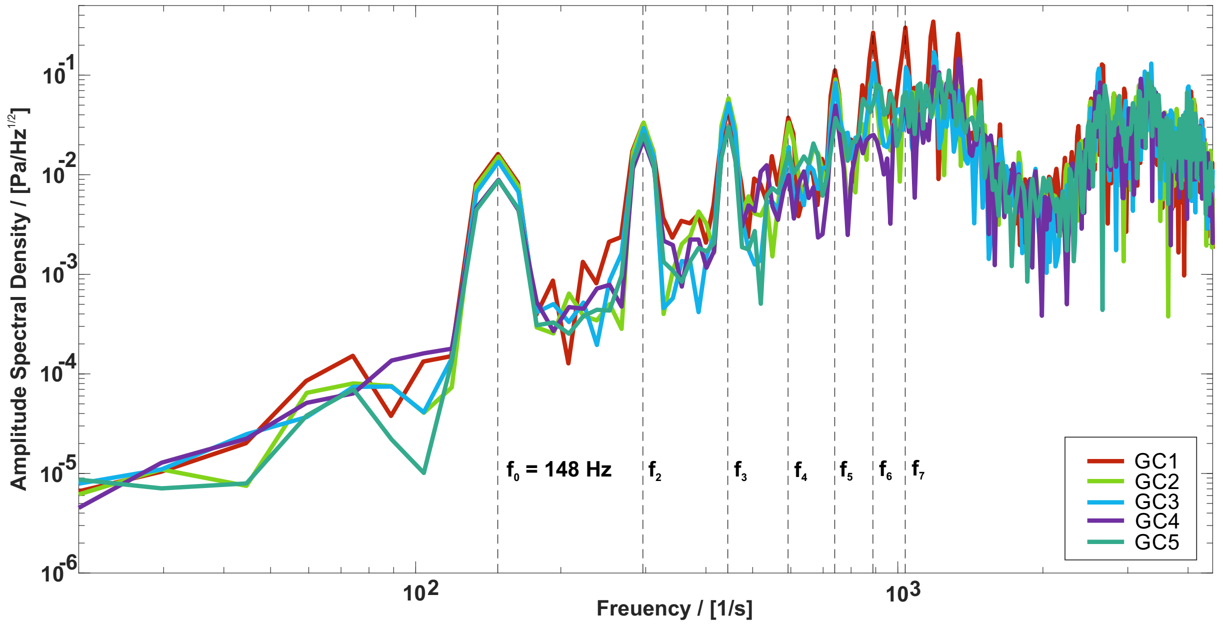

Figure S3. Amplitude Spectral Density (ASD) for the five GC type with asymmetric vocal fold motion. The spectra of the five GC types show similar slops and only slight deviations in the amplitudes at the fundamental frequency, whereas more significant differences at the higher harmonics occur.

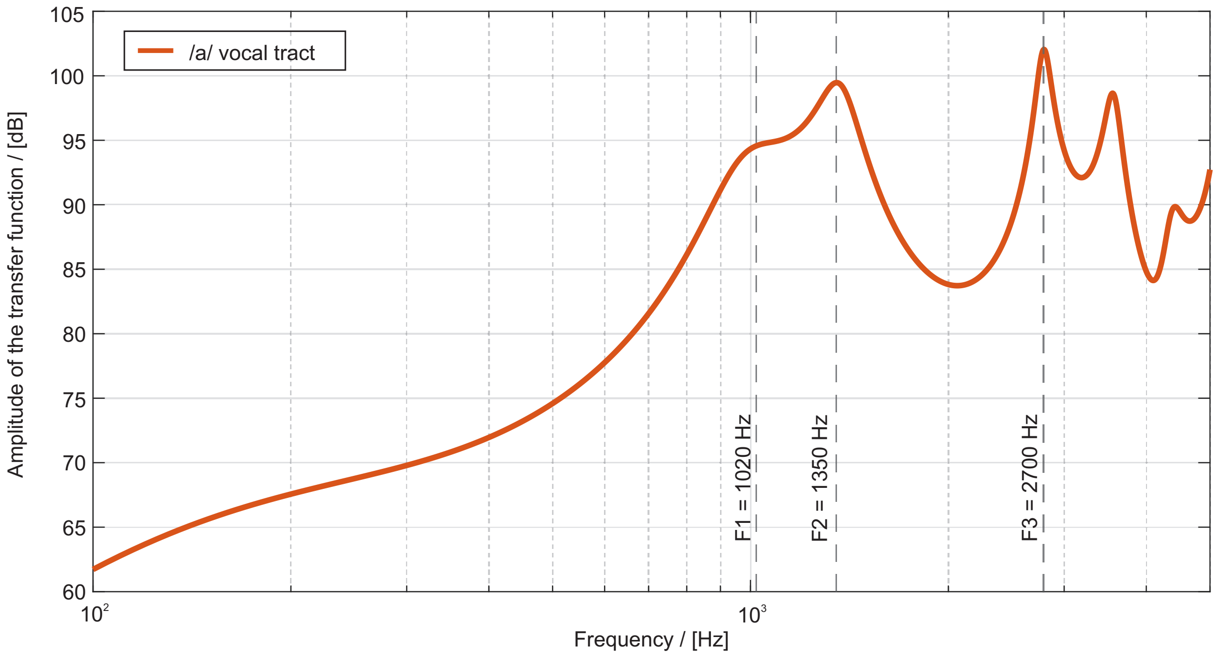

Figure S4. Transfer function of the /a/ vocal tract. The first three Formants are located at $F1 = 1020Hz$, $F2 = 1350Hz$, and $F3 = 2700Hz$.

Table S1. Summary of the meshes used for the grid resolution reduction study with the mean flow rate through the glottis $\overline{Q}$ and their relative deviations ($rel.Dev.$) in respect to the basis mesh MB.

Mesh	Base Size [mm]	Average number of cells	$\overline{Q}$ in $[\frac{l}{s}]$	$rel.Dev.$ to MB
MB	0.5	2.9 million	0.78	-
M1	0.56	2.1 million	0.78	0.0 %
M2	0.64	1.5 million	0.80	+2.6 %
M3	0.68	1.3 million	0.77	-1.3 %
